# On the Medical Topography of Malta

**Published:** 1831-04-01

**Authors:** 


### THE MEDICAL TOPOGRAPHY OF MALTA. By *John Hennen*, M.D.

> "Insula parva situ, sed rebus maxima gestis;
> Africæ et Europæ, ac Asiæ contermina, Pauli
> Hospes, et alborum procerum gratissima mater."
> *Vicenzo Littara in Abela.*

THE medical topography of Malta, by the late indefatigable Dr. Hennen, forms the principal part of his great work on the topography of the Mediterranean generally, and is by far the most finished specimen of this species of medical literature in the English language. Next to Gibraltar, the Island of Malta is the strongest and most important of all our possessions in the Mediterranean; and as it is now frequently recommended as a refuge for our invalids, its climate and locality become interesting objects of inquiry to medical practitioners in this country. On this account alone, we flatter ourselves that the following article, in which we have laboured to condense as much useful matter as possible in the smallest space, will not be deemed an unimportant portion of our present number.

This celebrated island, which has been claimed by Africa and Europe, lies between the two, and is about 60 miles in length by 35 in breadth. It contains neither rivers, lakes, nor morasses; but numerous *fiumari*, or beds of winter torrents, which are retentive of under-ground moisture, long after the surface has dried—besides several other sources of malaria. A principal locality of this kind lies at the head of the great harbour, and is called the "MARSA" or Marsh—another is the valley at the head of St. Paul's bay, "the marsh of Paoles"—and a third is seated at the head of the quarantine harbour—"MISSIDA." History, and even recent history, shews that these places have annually afforded the miasms that occasion intermittents in the human population.

The island is, generally speaking, low, the highest ground being little more than a thousand feet above the level of the sea. The surface, however, is beautifully diversified; and, were it planted with trees, would present a very picturesque line of hill and dale. A small range of craggy hills crosses the island, and is only influential in affording sources of spring water, and directing the course of the winter rains into the different valleys. These valleys generally run in a direction from South-West to North-East—and are supposed to be gradually filling up by the alluvial deposits. Were they not carefully drained, they would soon become very insalubrious.

### CLIMATE.

This has been variously represented by different writers. One thing is certain—that it is very healthy. In the year 1822, the maximum heat (within doors) was 90 degrees—the minimum 46—the medium 68°. In 1823, the medium was 69°.

"Every person accustomed to thermometrical observations is aware of the difference between sensible heat and that indicated by instruments. In Malta it is peculiarly striking, and greatly depends on the state of the winds; but it is at the night season that the heat is most oppressive, so much so, as to justify the term 'implacable,' which is often applied to it. The sun in summer remains so long above the horizon, and the stone walls absorb such an enormous quantity of its heat, that they never have a sufficient time allowed them to get cool; and, during the short nights, this heat radiates from them so copiously as to render the nights, in fact, as hot as the days, and much more oppressive to the feelings of those who are accustomed to associate the idea of coolness with darkness. I have seen the thermometer, in a very sheltered part of my house, steadily maintain, during the night, the same height to which it had arisen in the day, while I marked it with feelings of incalculably increased oppression; and this for nearly three successive weeks of August and September, 1822." 444.

Snow never falls at Malta. The rains fall with tropical violence in the months of December, January, and February. About March, the sky gets settled—and during June, July, and August, scarcely a cloud is seen. The rainy season is not particularly unhealthy—perhaps because the rains chiefly fall in the night, and are quickly absorbed by the chalky soil, and carried off by the well-drained streets. Winds from the South and West bring murky clouds, and envelop the island in fogs. The opposite winds sometimes bring with them dense fogs, and even heavy rains. The dews are extremely heavy in Spring and Autumn—and always when the sirocco blows. In the middle of Summer, they are rarely observed—and, at this season, the roofs of the houses, the balconies, and even the streets are the principal dormitories of the inhabitants, according to their respective ranks, and without injury to health, while moisture is avoided.

The most remarkable, if not the most prevailing winds, are from the S.E.—S. and N.W. The two former, the sirocco, are always damp, suffocating, and disagreeable—especially to the sick. The North-East wind called the "gregale," when moderate, is bracing and invigorating; but, when violent, the mercury sinks, heavy rains fall, and the sudden change of temperature is severely felt. There are no regular sea and land breezes at Malta. So soon as the sun sets, the atmosphere becomes close and sultry, and the breeze, if any, dies away. The volumes of impalpable dust which are rolled about at Malta, exert a considerable influence on health. The closest doors offer no effectual barrier to its entrance—the eyes are clogged—the fauces choaked—and the skin covered with it.

"When a damp wind blows, or when the dews and fogs are heavy, this floating dust is moistened, its particles coalesce, and it is precipitated from the atmosphere in the form of drops of a very fine mud; we frequently see the plants in a morning covered with this precipitate. It is something of this kind, unusually copious in quantity, which Dr. Maclean, in his 'Researches,' dignifies with the name of a 'shower of mud,' and which he conceives to have been a cause of the plague! This opinion is too absurd to require a serious refutation; the dust of Malta floats in the atmosphere for more than half the year; but although I do not admit of its producing pestilence, I have no doubt of its injurious effects on the eyes and lungs." 447.

Participating with Dr. H. in the absurdity of the *pestiferous* hypothesis, we cannot help thinking that the designation which Dr. Maclean gave this shower, was peculiarly appropriate.

The island is a rock of limestone, of different densities and species. The soil therefore cannot be supposed to be very deep or fertile. Nevertheless the climate is favourable to the production of tropical fruits and vegetables, and we find abundant supplies of these in the fields and markets. The principal vegetable productions are cotton, cummin, and oranges. The island does not produce more corn than would supply the inhabitants a third part of the year. Nothing like a wood or even grove exists on the island, and vegetation is, of course, on a very contracted scale. Perhaps it is owing to this very circumstance that the island is so comparatively salubrious. The population is estimated at 110,000 souls. The houses are built of very solid materials, and are well adapted to the climate. The roofs are flat, and covered with "terras," so as to be impenetrable by rain. The floors are generally of stone. Each house has a court, with a small well or tank; into this court many of the windows of the apartments open. The apartments are spacious, lofty, and cool in Summer; but, having no fire-places, are uncomfortable in Winter.

Fuel being scarce, its consumption is economized. A Maltese considers it unwholesome to heat his apartment by a fire—and so it is, where there is no fire-place. He therefore seeks for heat in warm clothing and well-closed doors and windows. Even the kitchens are scantily supplied with fuel, and immediately after the meal is dressed, the fire is extinguished, so that, during the remainder of the day—

> "Their kitchens vie in coolness with their grots."

The Maltese are frugal and abstemious, like all those who—

> "Force a churlish soil for scanty bread."

In regard to physical character, the natives of Malta stand in a respectable point of view. The men are about the middle size, erect and active. The women are below the average height, (and they have the Medicean Venus for a comfort,) but well formed—and many of them handsome. They suffer little in parturition, and generally leave their beds on the third day. They marry early. Many instances occur where females are mothers at the age of thirteen years.

### Endemic and Prevalent Diseases.

Remittents and intermittents are of annual occurrence among the resident inhabitants, and in considerable numbers. Although the sources of malaria are on a small scale, they are nevertheless quite adequate to the fevers which are seen of the type alluded to. Ophthalmia appears, in one form or other, every year.

"There is every reason to suppose, that those diseases principally proceed from the united influence of the excessive heat of the atmosphere, the great glare of the sun reflected from the rocks and numerous buildings, and the vast quantity of dust blowing about in all directions; while, during the night, the atmosphere is often particularly damp, from the copious depositions of dew, to the effect of which the lower orders are fully exposed, as they sleep frequently in the open air, and always with the head uncovered; in addition to which the sirocco winds of autumn are peculiarly damp of themselves." 496.

This is a more philosophic explanation than that which has been resorted to by the natives—namely, that ophthalmia is owing to the ripening of the pomegranates in Autumn—in the same way as they believe the activity of the fleas in early Summer is occasioned by the ripening of the beans!

Next to fever, the most prevalent diseases are bowel-complaints and *pulmonary affections.*

"The deaths by pulmonary affections are also very numerous; they are classed under six different heads in the bills of mortality, viz. cough, consumption, hæmoptysis, phthisis pulmonalis, pleuritis and pulmonic. If we even deduct the vague class of 'consumption,' we find the remainder very numerous, and the mortality occasioned by cough and phthisis pulmonalis alone, will of itself satisfactorily prove the great error of sending phthisical patients to this climate from England, under an idea that phthisis is rare, or continuing them here when their disease is once developed." 498.

Phthisis is rapid in its course, in this island, where, as in Italy, it is considered to be contagious, both by medical men and the inhabitants generally. Liver complaints are very numerous.

### PLAGUE.

The most important epidemic of Malta is the plague. The position of the island, so close to the Levant, must render it always exposed to the contagion of this dreadful disease; and nothing but the strictness of its quarantine, Dr. H. thinks, has preserved it from frequent visitations. The years of its occurrence, according to the most authentic accounts, were 1519—1593—1623—1663—1675—from which it had a respite till 1813, or 138 years!

How the contagion was introduced at the last-mentioned period, is a question still involved in doubt—"nothing amounting to positive certainty being known." The received opinion was, that it was imported in a polacre from Alexandria. Unfortunately for Malta, the nature of the disease was unsuspected until it had developed itself by destructive progress among the people, and bade defiance to all attempts at tracing the line of communication.

"It is in vain (says Dr. H.) to deny that many gross errors were committed at first, (both by the public authorities and the population,) from ignorance of the real nature of the disease, and from dread of the quarantine police; and it would be equally vain to attempt to conceal, that, after the disease was known to be plague, the best and most active measures were not at once adopted; but how often do we discover in the annals of the plague, that the cool promptitude which we are so ready to admire in theory is seldom exhibited in practice! It requires a rare combination indeed of knowledge, judgment, and activity, to cope with this insidious and deadly foe; and it is rarer still to meet with them all united in an individual who at the same time possesses the authority and vigour to bring them into full unembarrassed action. Such a man was he to whom Malta and Gozo owe the extinction of the plague which ravaged their shores in 1813 and 1814, and who, by a repetition of the same wise measures, was equally successful in other British possessions. It is with emotions of no ordinary kind, that I here insert an extract from a despatch addressed by him to Lord Bathurst, in April, 1819, a document which, I may venture to assert, displays throughout more solid information on plague, than has hitherto appeared even in the works of professional men. 'This is a subject,' says Sir Thomas Maitland, 'upon which I possibly have had more occasion to pay a very deep attention, than any other individual in his Majesty's service, and it is one upon which I can say, without flattering myself, I have had more practical experience than almost any other person, having arrived at Malta in the middle of the plague, and having since that witnessed the beginning and the end of three different plagues, one in the island of Gozo, one in the island of Corfu, and one in the island of Cephalonia.

'The subject itself is one of a most important nature; for there is no denying that the treatment of plague, under the ancient system, is one attended with a degree of cruelty and tyranny unparalleled in the annals of the world, and only to be defended on the principle of positive and ascertained necessity.

'This system cuts up by the root all those feelings of domestic life which are peculiarly endeared to the mind of man in a moment of sickness and distress; rends asunder all the usual bonds of society, and places the unfortunate patient in a situation of the most desolate isolation, at the moment when the only remaining comfort of life exists in the kindness of natural friends and connexions.

'The quarantine law, too, in the instance of the plague actually existing, is not only most arbitrary in itself, but to the full as indefinite as it is arbitrary; and the whole of the circumstances attached to it are so revolting to the feelings of every man, looked at in any way, that I apprehend that this is one of the principal reasons why, in almost every instance that can be mentioned, this fatal malady is allowed to arrive at a great height before it is even declared to be plague; and in the two great instances of the plague at Messina and Marseilles, we accordingly find that no reliance was placed on its being the plague till it got to that dreadful head that occasioned those miserable scenes which afterwards ensued.

'The same was considerably the case at Malta at the breaking out of the plague, and it cannot be astonishing to any man who has seen it, that even the last dregs of hope must expire, before any society can submit patiently to a system of discipline which can be stated at best as only an inferior evil to the plague itself.

'The quarantine laws under the same system, with the view to prevent the introduction of plague, are attended, in all instances, with evils of great magnitude, and most of all, in the very serious effects it universally has upon the commercial relations of different countries. It would be most fortunate indeed, if it could be made out that the world had hitherto been mistaken with regard to its causes and its origin.'" 505.

In most of the plague countries this dreadful malady spontaneously subsides at the Summer solstice; but in Malta this fortunate trait in the character of the disease did not appear. We shall not follow Dr. Hennen in his account of the symptomatology of the plague. No medical man on the island met with a second occurrence of the disease in the same individual; yet instances of this are on record. Our author is a firm believer in the contagious nature of plague, though he acknowledges that season, climate, and indigenous causes have great influence over the disease. The following passage is interesting.

"I shall now give an instance of the complete immunity afforded by separation, where the distance between the infected and the sound was reduced to a few feet, and where the contagion was literally at their very doors. I have already observed that the lower parts of the houses of Valetta are generally occupied as shops, stores, &c., or places of residence for the poorer classes of inhabitants. In the lower part of the hospital of one of the regiments quartered in Valetta during the plague, there resided no less than seven distinct families in an equal number of separate habitations. The plague raged among these unfortunate people to such an extent, that four families were totally cut off, and of the remainder little more than a fourth escaped with life: the plague raged in every corner of these dwellings, and yet the hospital, which was situated on the upper floor, but whose inmates were kept under the strictest control, escaped all contamination whatever. The heated air and exhalations from the habitations below were in no way guarded against, but had unimpeded access to the windows of the upper apartments, towards which they must naturally have ascended at all times from their greater levity, and into some of which every wind that blew must have impelled them in a state of great concentration. In front of this hospital, it is true, there is a small square or open space, and on one side runs a street (Strada Ponente) of nearly the ordinary breadth; but in the rear it joins a large mass of buildings, and on the western face there is a narrow blind alley only ten feet wide, into which the doors of all the contaminated houses opened, and as these doors were the only means of communication with the external atmosphere, the pent-up space must have been continually filled with the accumulated exhalations from the diseased. This was not a solitary instance of the residents on one floor of a house enjoying the reward of proper precautions, while their negligent neighbours above, below, and around them, were falling victims to the plague; but it is rendered remarkable, when we recollect how susceptible to all impressions from atmospheric influence, and from disease of every description, the residents of an hospital usually are, under even the ordinary circumstances of military practice." 513.

Dr. H. next adverts to a well-known case, in which plague actually existed within the walls of a building, and yet was prevented from spreading by strict separation. The convent of St. Augustine was the scene of this segregation and security. From the first alarm, the Padre Maestro Valla, principal of the convent, maintained the utmost caution in communicating with the public; but at length a servant purchased some old clothes in the Mandraggio, and was soon taken ill, together with one of the monks who attended on him. They were both put in strict quarantine in their respective cells—and the convent was converted by the activity or fears of the Padre, into a well-organized lazaretto. The servant and his attendant both died of plague, but no one else in the building caught the contagion.

The most remarkable instance, however, of the efficacy of insulation, and the inability of the pestilent miasma to propagate itself through the medium of the air, occurred at Casal Curmi, a village in one of the valleys of the island. The inhabitants became extensively affected with the plague, and the governor determined to draw a line of circumvallation, by means of a cordon of troops, around this ill-fated village. The consequence was, that no disease radiated from this focus of contagion, not even to the soldiers who guarded it.

It is unnecessary to go into the details of the specific prophylactic measures that have been proposed and practised against this terrible visitation—such as oily inunctions, the crape mask, fumigations, tobacco leaves between the fingers of the attendant and the wrist of the patient. We have little confidence in any of them. The oily frictions appear to have most facts in their favour; but the profuse perspirations which they occasioned prevented them being regularly employed. We entirely agree with our intelligent author in the following passage.

"The best plan appears to me to be, first, the free and continual admission of air to all parts of the house and furniture, the removal of filth of every species, ablution of all the wood-work, by a strong lye of soap and water, and the application of hot lime-wash to all the walls, from the cellar to the garret; taking care to remove and repair all loose or decayed pieces of plastering. All the drains, necessaries, &c., should be thoroughly cleansed, which may be well done after the grosser matters are removed, by pouring down them the water of the tanks, which, as well as all other reservoirs, should be completely emptied of their contents, in order to undergo a thorough cleansing. The clothing and furniture should be most minutely cleansed, and such parts as are not susceptible of damage from water should be submitted to copious affusions and even boiled in a strong lye when practicable." 533.

"Upon the whole, the history of the plague of Malta furnishes us with the strongest arguments in favour of the theory of contagion, and justifies, in the most ample manner, the practice of those, who place their sole dependence on prompt and decisive measures of police, persevered in long after the exigencies which first rendered them necessary, may, to the inexperienced eye, have appeared to cease.

Medicine, generally speaking, is of little avail; but however useful the rules of the physician may be found, under certain circumstances, the old distich should never be forgotten, which, though trite, contains the correct rule of conduct:—

"Hoc tria labificam tollunt adverbia pestem
"Mox, Longe, Tarde, cede, recede, redi." 535.

### LONGEVITY.

In a hot climate, like Malta, the external indications of age increase rapidly, "and often give to maturity, and even to youth, the appearance of a much greater advance in life than they have really made." Nevertheless there are instances on record, though we may suppose them rare, where people have lived to the advanced age of 90, 100, and even 110 years. Dr. Hennen could not ascertain the mean duration, or the value of life, which is of far more consequence than a few rare examples of longevity, which are merely exceptions to general rules.

The only subject which we shall notice is that of—

### PULMONARY AFFECTIONS.

Dr. H. takes the pulmonary diseases as he finds them in the military returns, and consequently it cannot be expected that the nosological designations should be always accurate. From these returns, as well as those collected in the Ionian Islands, Dr. H. considers it all but proved that malarious situations are those most free from pulmonary affections in general. From the table of admissions in Malta it appears that pulmonary complaints were in the proportion of about one to eleven of all other complaints put together. It is to be remembered that soldiers are not enlisted or sent to a foreign climate labouring under any symptom of phthisis or other dangerous disease —that they are, generally speaking, in the prime of life—that they are disciplined and kept in order—and that they have prompt and excellent medical attendance. These circumstances taken into consideration, we may well conclude that Malta is not the place for phthisical invalids. Indeed Dr. H. expressly informs us that all men who presented symptoms of this disease were sent home to England by every possible conveyance.

"I would observe," says Dr. Hennen, "that heat, such as is experienced at Malta, produces a great 'plethora ad volumen,' and determination of blood to particular internal parts, especially those weakened by previous disease; I would also observe, that frequent alternations, such as occur at Malta, must greatly affect the regularity of pulmonary exhalation; but I conceive that there is another powerful effect of the atmosphere to be considered.

Saussure, Humboldt, and others, who have ascended high mountains, have constantly found that, as the air has become rarefied, their respiration has become impeded, pains in the chest have occurred, and, in several instances, hemorrhages from the lungs and nose have taken place, so as to oblige them to descend. Now, in Malta, we have a rarefaction of the air, not, indeed, from the same cause, nor to the same extent that is observed on the tops of mountains, but sufficient to affect the sensations very strongly in breathing, especially when that function is constantly performed under its influence. But it may be said, why does not the rarefaction of the air equally affect the lungs in the Ionian islands, where the heat is nearly as great, and where it is admitted to be more disagreeable to the feelings? The answer, I conceive, is very satisfactorily furnished; the moisture of the air in the islands obstructs its great rarefaction; while, in Malta, it is exsiccated to the highest degree from the general aridity of the soil." 604.

Although we do not subscribe to Dr. Hennen's reasonings respecting the unfavourable operation of a Maltese climate on pulmonary complaints, yet we have no doubt as to the fact. No one, indeed, who navigated the Mediterranean seas, or traversed its islands and surrounding shores—and who, at the same time, was competent to judge, could possibly infer that such localities were favourable to diseases of the chest. It is very true that, in Italy, Greece, and the Islands of the Mediterranean, but especially in Italy, the vicissitudes of temperature, and of drought and moisture, are not so *frequent* as in England and some other countries; but then they are equally as sudden—and to a far *greater extent.* It is the *infrequency* of occurence, and the *immense range* of the transition, when it does occur, that renders the said transition so detrimental under the boasted skies of Italy. The reason must be obvious to every medical capacity. The human frame becomes *habituated* to atmospherical impressions, when they are moderate and repeated at short intervals—but not so when they are *immoderate,* and with long intervals between them.*

We have now concluded our review of Dr. Hennen's work, which we have divided into different articles, in order that we might be able to exhibit a clearer view of each particular subject. The profession has long ago decided on the merits of the industrious and intelligent author, so that eulogy is now superfluous. An acquaintance of nearly twenty years with the deceased—and not merely an acquaintance, but a cordial and sincere friendship, strongly urge us to give vent to our feelings on taking a final adieu of Dr. Hennen and his works! But alas!

"Can honour's voice provoke the silent dust,
"Or flattery soothe the dull cold ear of death?"

It is not probable that the departed spirit is permitted to know what goes on after the tomb has closed over its mortal fabric; and therefore it is useless to say any thing that would have been personally pleasing, before the debt of Nature was paid! The best thing we can do for the dead and the living, is to give every diffusion in our power to the useful labours of him who has ceased to toil and to feel for the good and for the sufferings of his survivors.

REQUIESCAT IN PACE!

---

* See the Editor's observations on the subject in a work just published, entitled "CHANGE OF AIR, OR AUTUMNAL RELAXATION."

